# SENP7 knockdown inhibited pyroptosis and NF-κB/NLRP3 inflammasome pathway activation in Raw 264.7 cells

**DOI:** 10.1038/s41598-020-73400-w

**Published:** 2020-10-01

**Authors:** Xun Li, Fangzhou Jiao, Jia Hong, Fan Yang, Luwen Wang, Zuojiong Gong

**Affiliations:** 1 Department of Infectious Diseases, Renmin Hospital of Wuhan University, Wuhan, 430060 China; 2 Department of Obstetrics and Gynaecology, Renmin Hospital of Wuhan University, Wuhan, 430060 China

**Keywords:** Inflammasome, Molecular medicine

## Abstract

Pyroptosis is a kind of necrotic and inflammatory programmed cell death induced by inflammatory caspases. SENP7 is a SUMO-specific protease, which mainly acts on deconjugation of SUMOs from substrate proteins. We evaluated the effect of SENP7 knockdown on pyroptosis, NF-κB signaling pathway, and NLRP3 inflammasome in Raw 264.7 cells. The results showed that the GSDMD protein mainly expressed in the cytoplasm nearby nuclei of Raw 264.7 cells. It migrated to cytomembrane with the numbers of Raw 264.7 cell decreased when LPS + ATP were administrated. Which was inhibited by SENP7 knockdown. In addition, not only the pyroptosis of Raw 264.7 cells was inhibited, the activation of NF-κB signaling pathway and NLRP3 inflammasome were also attenuated by SENP7 knockdown. The mechanism may be associated with the over SUMOylation of proteins induced by SENP7 knockdown.

## Introduction

Pyroptosis is a form of regulated cell death (RCD) with specific morphological features including cell swelling and a specific form of chromatin condensation, culminating with plasma membrane permeabilization^1,2^. In contrast to apoptosis, pyroptosis is a kind of necrotic and inflammatory programmed cell death induced by inflammatory caspases^3^. It is regulated by caspase-1 dependent or caspase-1 independent mechanisms. In caspase-1-dependent manner, also known as canonical inflammasome activation, Caspase-1 is activated by an inflammasome initiation sensor that recognizes either cause-associated or risk-associated molecular patterns^3^. Caspase-1-independent pyroptosis is activated by noncanonical inflammasome activation, caspase-4, caspase-5 and caspase-11 are the apical activators in this mode^3^.

The activation of inflammasome plays a key role in pyroptosis course. Among the inflammasomes, NLRP3 inflammasome is the most widely characterized. Activation of NLRP3 leads to recruitment of the adapter apoptosis-associated speck-like protein, resulting in activation of the precursor of caspase-1 to caspase-1^4,5^. Caspase-1 then promotes the conversation of pro-IL-1β and pro-IL-18 into IL-1β and IL-18, and initiates pyroptosis^4^.

SUMOylation is an important posttranslational protein modification that regulates the stability, activity and functions of proteins^6^. The course of protein SUMOylation is dynamic and reversible, which is regulated by E1 enzyme, E2 conjugating enzyme Ubc9, E3 ligases and SUMO-specific proteases (SENPs)^7^. It is reported that SUMOylation regulates NLRP3 inflammasome activation^8^. But whether pyroptosis is also regulated by SUMOylation is still unknow. SENP7 is a SUMO‐specific protease, which mainly acts on deconjugation of SUMOs from substrate proteins. In this study, we construct a stably transfected cell line of SENP7 knockdown to inhibit its deSUMOylation and to evaluate the effect of SENP7 knockdown on the pyroptosis, NF-κB signaling pathway and NLRP3 inflammasome.

## Materials and methods

### Cell culture

The murine macrophage cell line Raw 264.7 was purchased from the Cell Bank of the Chinese Academy of Sciences (Shanghai, China), and cultured in dulbecco's modified eagle medium (DMEM) supplemented with 10% fetal bovine serum, 20 U/ml penicillin and 20 μg/ml streptomycin in an incubator at 37 ℃ with 5% CO_2_ under a humidified atmosphere.

### Establishment of SENP7 stable knockdown Raw 264.7 cells

Three siRNA sequences (522, 440 and 2882) targeting SENP7 gene were designed and the most effective sequence (440) was screened out. After lentiviral expression vector pLVX-shRNA2-SENP7 carrying the siRNA sequence (440) was constructed, it was transfected into 293 T cells and packed into pLVX-shRNA2-SENP7 lentivirus. Raw 264.7 cells were then transfected with the pLVX-shRNA2-SENP7 lentivirus. Stably transfected cells were screened out for the next experiments. The siRNA sequences were listed as below: Senp7-mus-522, sense 5′-CGA CAU CUG UUG ACA GCA UTT, anti-sense 5′-AUG CUG UCA ACA GAU GUC GTT-3′; Senp7-mus-440, sense GGA CGA GAA UUC AGA AAG ATT-3′, anti-sense 5′-UCU UUC UGA AUU CUC GUC CTT-3′; Senp7-mus-2882, sense 5′-GCG GCC AUG UAU UCU CAU ATT-3′, anti-sense 5′-UAU GAG AAU ACA UGG CCG CTT-3′.

### Induction of pyroptosis

Raw 264.7 cells with or without shRNA intervention were administrated with LPS (1 μg/ml) + ATP (5 mmol/L) for 24 h to induce pyroptosis^8^. The supernatants and cells were then collected for the following detection.

### Detection of pyroptosis

The pyroptosis of Raw 264.7 cells was tested by immunofluorescence assay. Briefly, cells were grown on glass coverslips, washed thrice with PBS for 3 min, fixed with PBS containing 4% formaldehyde for 15 min, and permeabilized with 0.5% Triton X-100 for 20 min. After washing with PBS thrice for 3 min, cells were incubated overnight at 4 ℃ with the antibody against GSDMD (Abcam, UK). They were then washed with PBS and incubated with Cy3-conjugated goat anti-rabbit Ig G (Beyotime Biotechnology, China). Nuclei were stained with DAPI (Beyotime Biotechnology, China). Finally, coverslips were observed using a fluorescence microscope.

### The inflammatory cytokine ELISA

Levels of IL-1β and IL-18 in Raw 264.7 cell culture supernatants were determined using high sensitivity ELISA kits (eBioscience, Austria), as per the instructions of the manufacturer.

### Quantitative real-time polymerase chain reaction (qPCR)

Total RNA was extracted from the Raw 264.7 cells and reverse transcribed using PrimeScript RT reagent kit (Takara Bio Inc., Japan). According to the manufacturer’s instructions, RT-PCR was performed with cDNA using gene-specific primers, the SYBR Green kit (Takara Bio Inc., Japan) and a 7500 Sequence Detector system (Applied Biosystems). The primers which developed with Primer Express software (Applied Biosystems) were listed as below. Then gene expression was normalized using the threshold cycle of the housekeeping gene *β-actin*. The primers used in this study: *β-actin*, forward 5′-CAC GAT GGA GGG GCC GGA CTC ATC-3′, reverse 5′-TAA AGA CCT CTA TGC CAA CAC AGT-3′; *senp7* forward 5‘-ATG GAC GGA CTT AGG ACG AG-3′, reverse 5′-AAC TCT GGG TGT CCT TTC GG-3′.

### Western blot assay

Cells were lysed and total proteins were subjected to sodium dodecyl sulfate–polyacrylamide gel electrophoresis (SDS-PAGE), transferred to a polyvinylidene difluoride membrane (Millipore, Germany). The membrane was sequential incubated with primary antibody and secondary antibody (LI-COR Co., USA), then detected by ODYSSEY infrared imaging system (LI-COR Co. USA). The primary antibodies including caspase-1 (#24232), cleaved-caspase-1(#89332), IL-1β (#12242), NF-κB P65 (#8242), phospho-NF-κB p65 (#3033), IκBα (#4814), phospho-IκBα (#2859), cleaved-GSDMD (#50928), TNF-α (#11948) and IL-6 (#12912) were purchased from Cell Signaling Technology, USA; GSDMD (ab239377), IL-18 (ab71495) and NLRP3 (ab214185) antibodies were purchased from Abcam, UK.

### Statistical analysis

Statistical analysis was performed using SPSS software (version 19.0). Data are presented as the mean ± SD. The differences between two groups were made using Student’s *t* test. Multiple groups were determined by one-way analysis of variance (ANOVA). A probability value of 0.05 or less was considered statistically significant.

## Results

### Lentiviral-delivered shRNA downregulates SENP7 expression in Raw 264.7 cells

**Figure 1:**
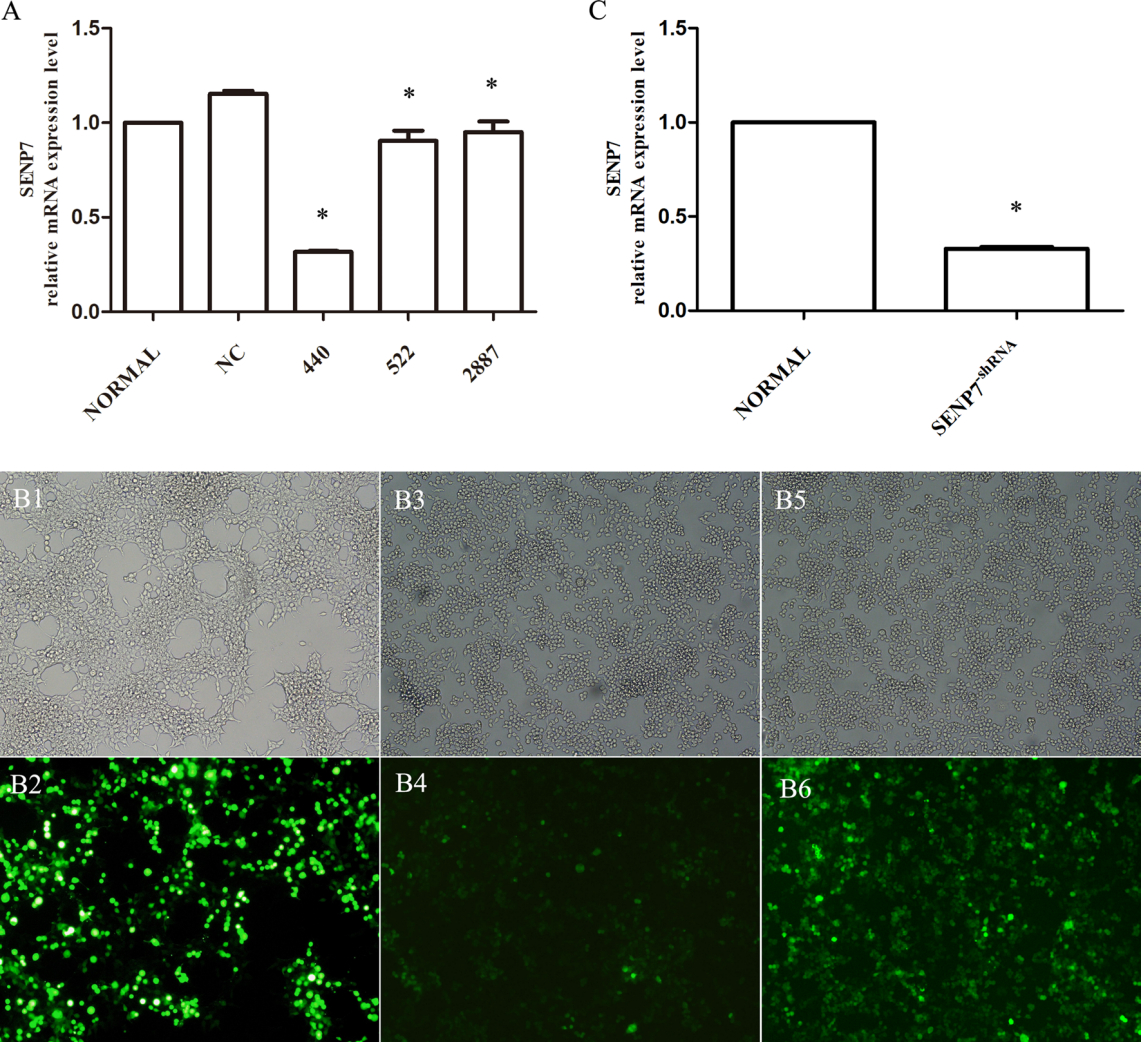
Establishment of SENP7 stable knockdown Raw 264.7 cells. (**A**) The Raw 264.7 cells were interfered by three siRNA sequences (522, 440 and 2882) targeting SENP7 gene. The expression levels of SENP7 mRNA were detected by Quantitative RT-PCR. NORMAL: Raw264.7 cells without any treatment; NC: Raw264.7 cells interfered with empty vector; 440: Raw 264.7 cells interfered with siRNA sequence 440; 522: Raw 264.7 cells interfered with siRNA sequence 522; 2887: Raw 264.7 cells interfered with siRNA sequence 2887. (**B1**–**B6**) Construction and screening of SENP7 stable knockdown Raw 264.7 cells. (**B1**,**B2**) 293 T cells were transfected with pLVX-shRNA2-SENP7 carrying the siRNA sequence (440). (**B3**,**B4**) Raw 264.7 cells were infected with the pLVX-shRNA2-SENP7 lentivirus. (**B5**,**B6**) SENP7 stable knockdown Raw 264.7 cells (Raw264.7-SENP7^shRNA^ cells) were screened out. (**C**) The SENP7 mRNA expression levels in Raw 264.7-SENP7^shRNA^ cells. NORMAL: Raw264.7 cells without any treatment, SENP7^shRNA^: SENP7 stable knockdown Raw 264.7 cells. Compared with the NORMAL group, *p < 0.05.

In the present study, three siRNA sequences targeting SENP7 gene were designed and applied to interfere Raw 264.7 cells. Senp7 mRNA levels were measured by quantitative RT-PCR. The results showed that 440 was the most effective siRNA sequence. After lentiviral expression vector pLVX-shRNA2-SENP7 carrying the siRNA sequence was constructed. It was transfected into 293 T cells and packed into pLVX-shRNA2-SENP7 lentivirus. Raw 264.7 cells were then transfected with the pLVX-shRNA2-SENP7 lentivirus. Stably transfected cells (SENP7^shRNA^) were screened out for the next experiments. The SENP7 expression in Raw 264.7 cells and Raw 264.7-SENP7^shRNA^ cells were detected by quantitative RT-PCR. The results showed that the SENP7 expression was significantly decreased in the SENP7^shRNA^ group than that in the normal group (about 1/3 compared to the normal group) (Fig. 1C).

### SENP7 knockdown inhibits pyroptosis in Raw 264.7 cells

**Figure 2:**
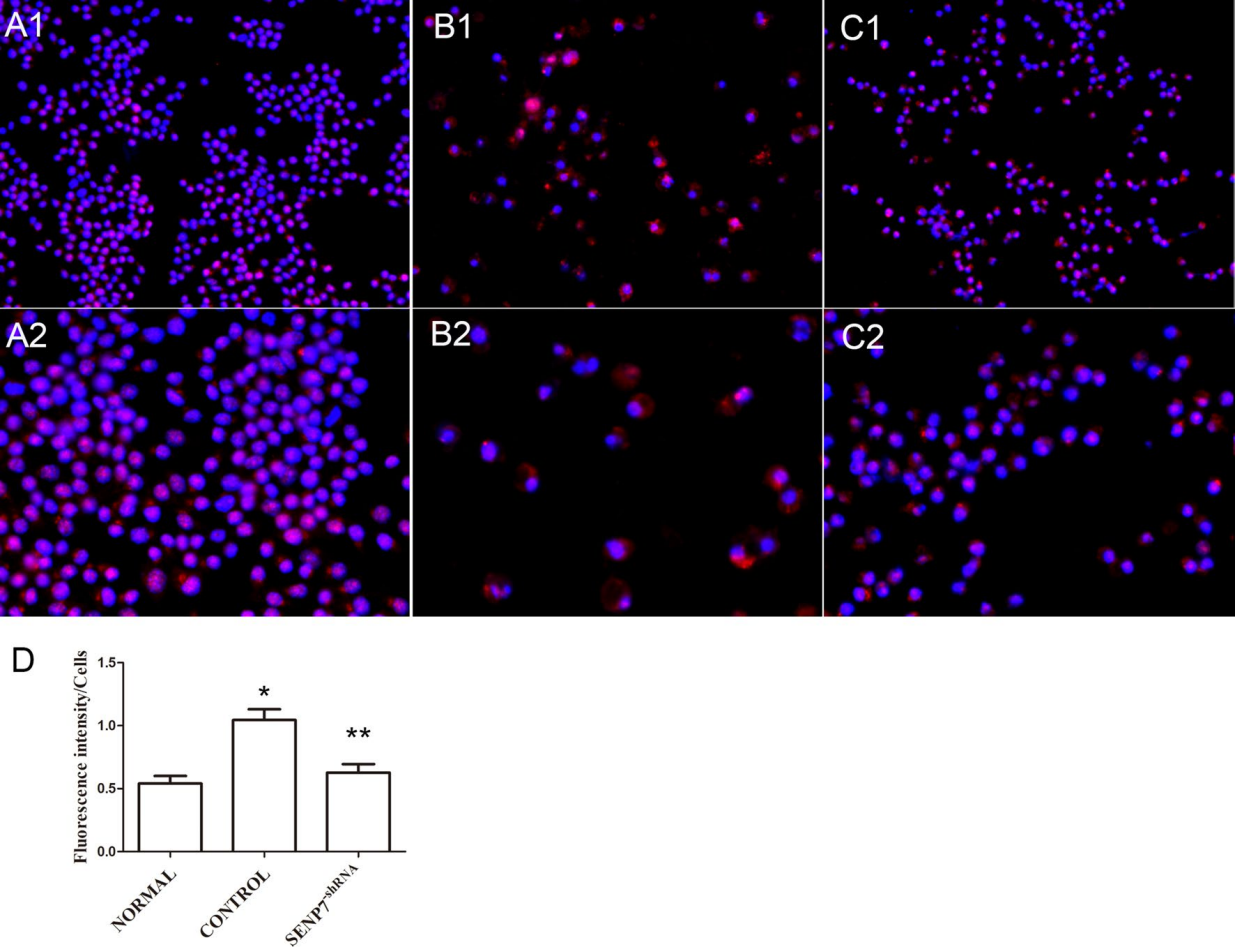
The GSDMD expression detected by immunofluorescence assay. The protein expressions of GSDMD were analyzed by immunofluorescence assay. GSDMD was probed by GSDMD antibody, The nucleus was stained by DAPI. (**A1**,**A2**) Raw 264.7 cells without any treatment, (**A1**) (× 200), (**A2**) (× 400); (**B1**,**B2**) Raw264.7 cells treated with LPS + ATP; (**C1**,**C2**) SENP7 stable knockdown Raw 264.7 cells treated with LPS + ATP. (**A1**,**B1**,**C1**) (× 200), (**A2**,**B2**,**C2**) (× 400). (**D**) The relative protein expression levels of GSDMD from three independent pictures were represented as the density of GSDMD fluorescence intensity normalized to numbers of cells. Compared with the NORMAL group, *p < 0.05, compared with the CONTROL group, **p < 0.05.

**Figure 3:**
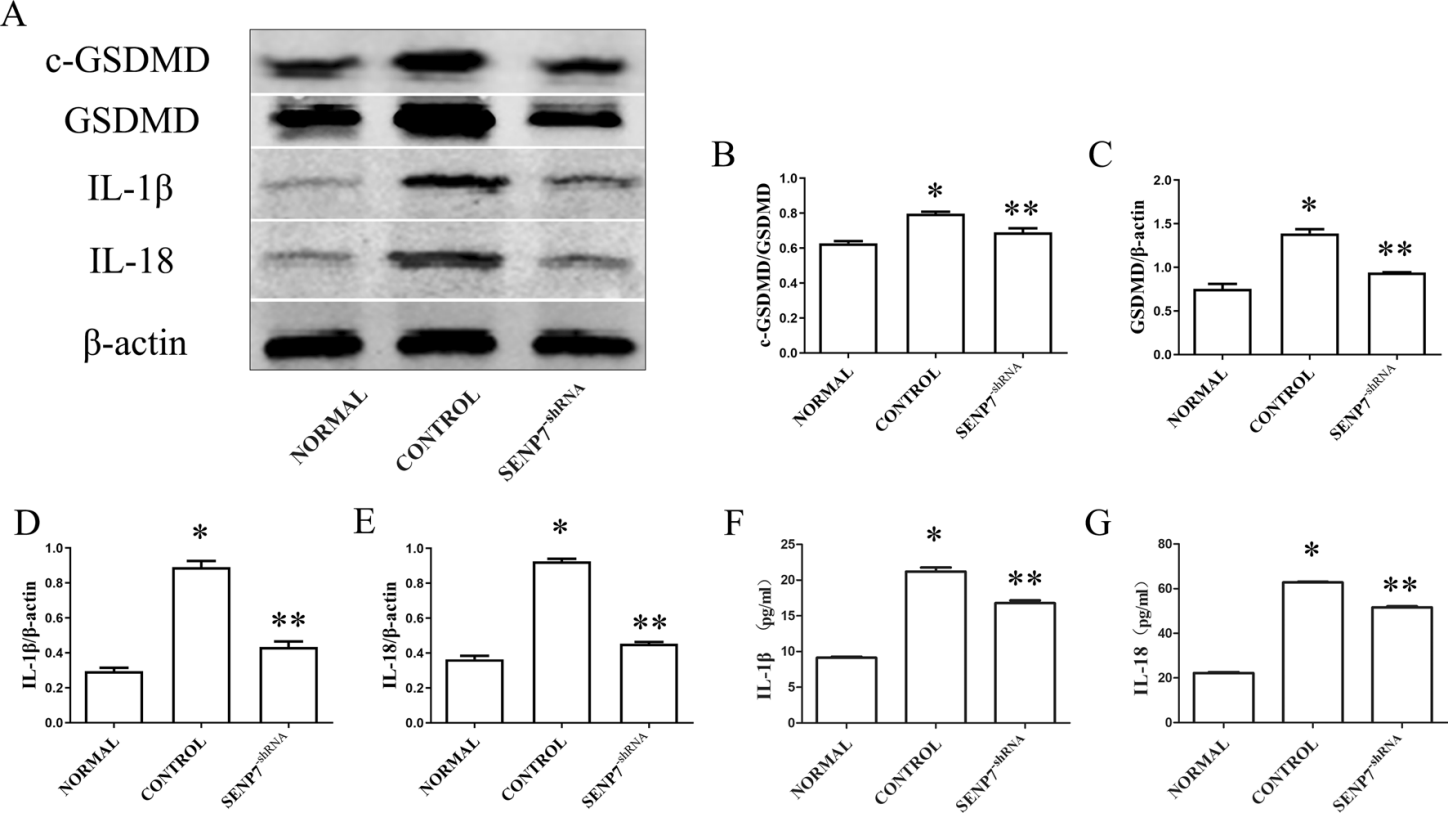
SENP7 knockdown inhibits pyroptosis in Raw 264.7 cells. (**A**) Cleaved-GSDMD, GSDMD, IL-1β and IL-18 expression levels detected by western blot assay; (**B**–**E**) The relative protein expression levels from three independent experiments are represented as the density of Cleaved-GSDMD, GSDMD, IL-1β and IL-18 bands normalized to that of GSDMD or β-actin bands. (**F**,**G**) The expression levels of IL-1β and IL-18 in supernatants detected by ELISA assay. NORMAL: Raw 264.7 cells without any treatment; CONTROL: Raw 264.7 cells treated with LPS + ATP; SENP7^-shRNA^: SENP7 stable knockdown Raw 264.7 cells treated with LPS + ATP. Compared with the NORMAL group, *p < 0.05, compared with the CONTROL group, **p < 0.05.

LPS + ATP were administrated to the Raw 264.7 cells to induce pyroptosis. The GSDMD protein was detected by immunofluorescence assay. The result showed that the GSDMD protein mainly expressed in the cytoplasm nearby nuclei of Raw 264.7 cells in the normal group. It migrated to cytomembrane with the numbers of Raw 264.7 cell decreased, when LPS + ATP were administrated (Fig. 2A1, A2, B1, B2). The phenomenon was inhibited by SENP7 knockdown (Fig. 2C1, C2). The expression levels of cleaved-GSDMD, GSDMD, IL-1β and IL-18 in Raw 264.7 cells were detected by western blot assay. The results showed that the expression levels of cleaved-GSDMD, GSDMD, IL-1β and IL-18 increased significantly in the control group compared to the normal group. But they were decreased in the SENP7^shRNA^ group compared to the control group (Fig. 3). The expression levels of IL-1β and IL-18 in the supernatants were also detected by ELISA assay. The results showed that the IL-1β and IL-18 expression levels were decreased in the SENP7^shRNA^ group than those in the control group (Fig. 3F, G).

### SENP7 knockdown inhibits NLRP3 activation in Raw 264.7 cells

**Figure 4:**
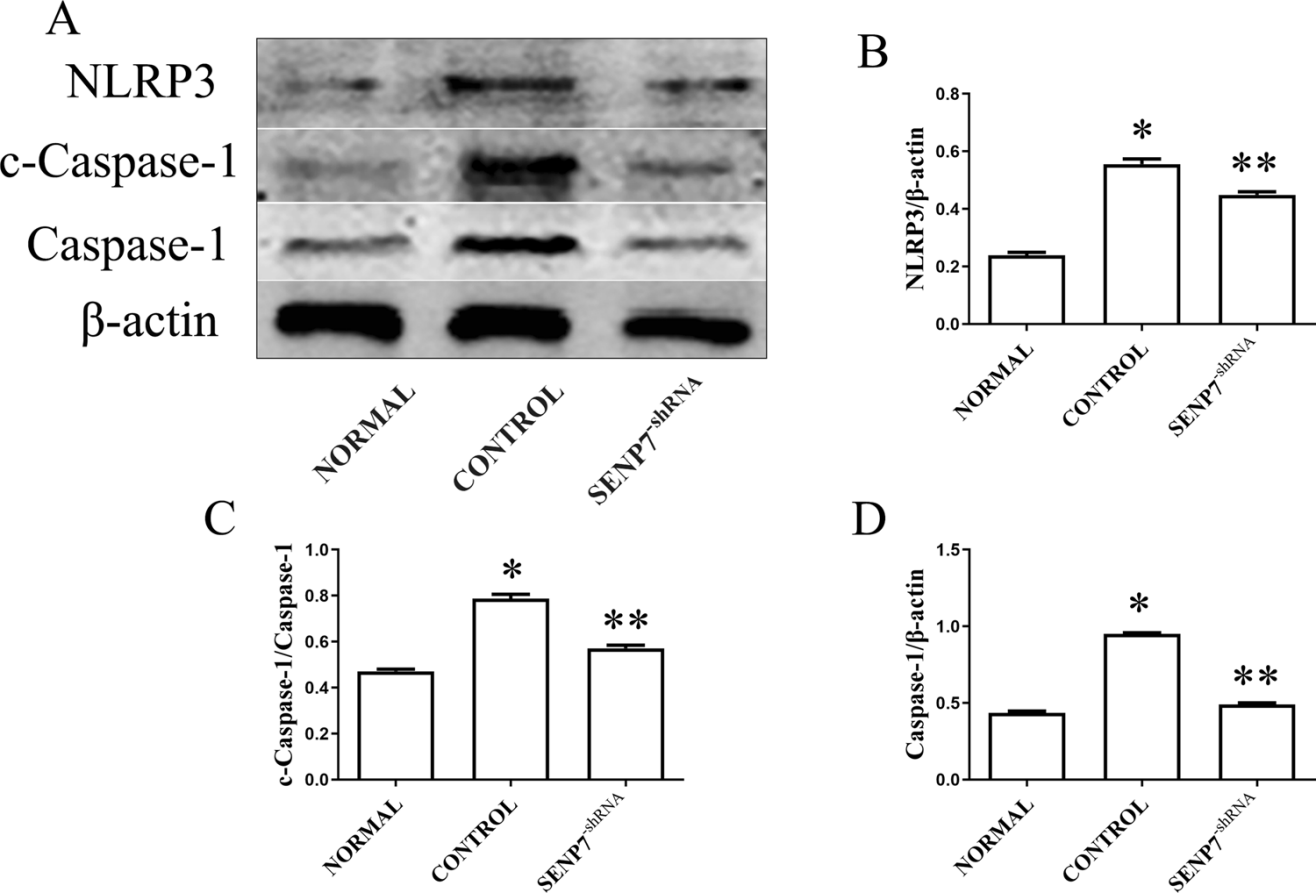
SENP7 knockdown inhibits NLRP3 activation in Raw 264.7 cells. (**A**) The expression levels of NLRP3, caspase-1 and cleaved-caspase-1 detected by western blot assay. (**B**–**D**) The relative protein expression levels from three independent experiments are represented as the density of NLRP3 and Caspase-1 bands normalized to that of β-actin bands or the density of cleaved-caspase-1 bands normalized to that of caspase-1 bands. NORMAL: Raw 264.7 cells without any treatment; CONTROL: Raw 264.7 cells treated with LPS + ATP; SENP7^-shRNA^: SENP7 stable knockdown Raw 264.7 cells treated with LPS + ATP. Compared with the NORMAL group, *p < 0.05, compared with the CONTROL group, **p < 0.05.

To evaluate the effect of SENP7 knockdown on the NLRP3 inflammasome, the expressions of NLRP3, caspase-1 and cleaved-caspase-1 were detected by western blot assay. The results showed that the expression levels of NLRP3, caspase-1 and cleaved-caspase-1 were lower in the SENP7^shRNA^ group than those in the control group (Fig. 4).

### SENP7 knockdown inhibits the activation of NF-κB signaling pathway in Raw 264.7 cells

**Figure 5:**
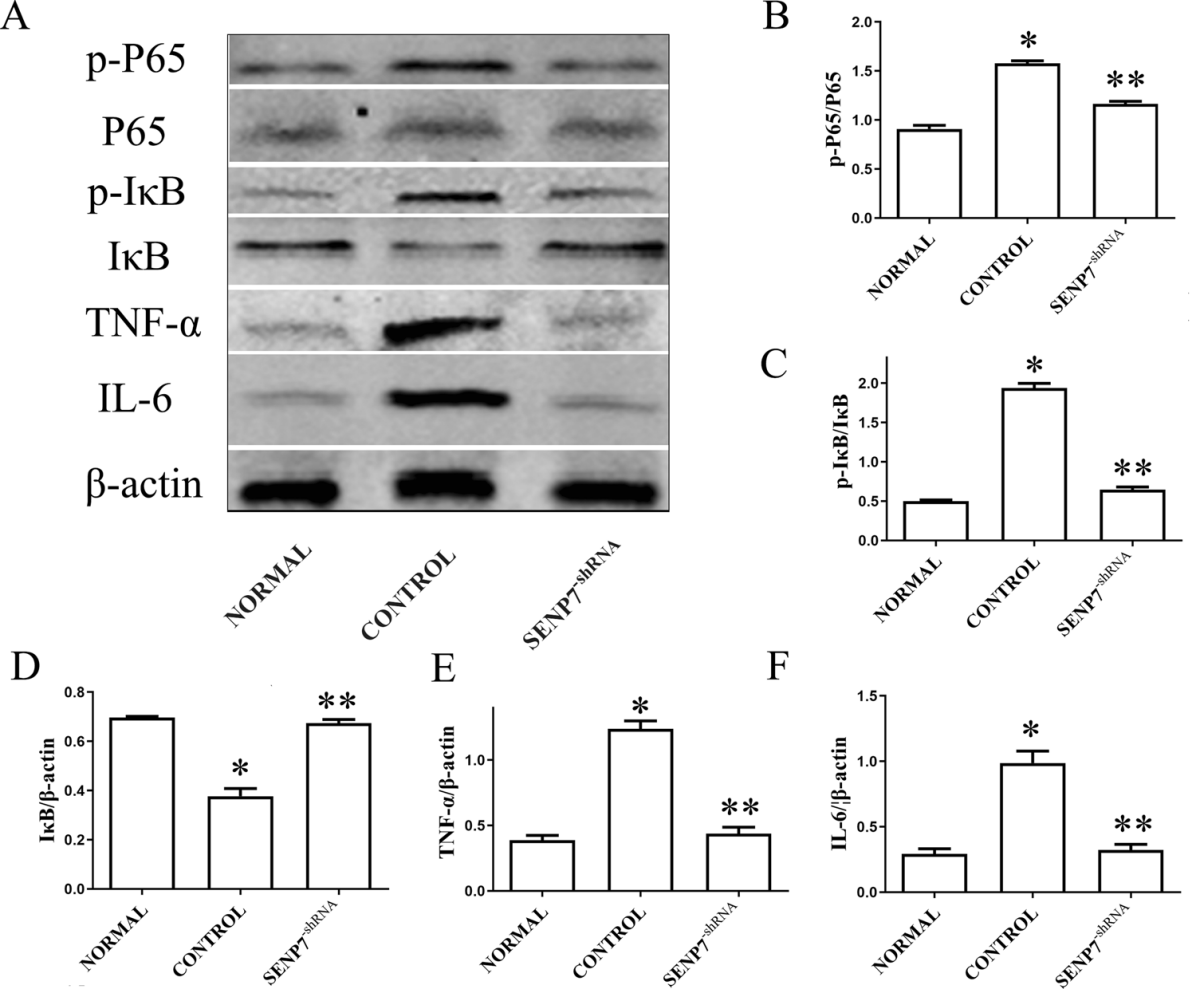
SENP7 knockdown inhibits NF-κB activation in Raw 264.7 cells. (**A**) The expression levels of NF-κB P65, Phospho-NF-κB p65, IκBα, phospho-IκBα, TNF-α and IL-6 were detected by western blot assay. (**B**–**F**) The relative protein expression levels from three independent experiments are represented as the density of target bands normalized to that of NF-κB P65, IκBα or β-actin bands. NORMAL: Raw 264.7 cells without any treatment; CONTROL: Raw 264.7 cells treated with LPS + ATP; SENP7^-shRNA^: SENP7 stable knockdown Raw 264.7 cells treated with LPS + ATP; p-P65: phospho-NF-κB p65; P65: NF-κB P65. Compared with the NORMAL group, *p < 0.05, compared with the CONTROL group, **p < 0.05.

The effect of SENP7 knockdown on NF-κB signaling pathway was detected by western blot assay. The results showed that the expression level of IκBα were higher in the SENP7^shRNA^ group than that in the control group, while the expression levels of TNF-α, IL-6, phosphorylation of NF-κB P65 subunit and phospho-IκBα were all decreased by SENP7 knockdown (Fig. 5).

## Discussion

SUMOylation is an enzymatic cascade which leading to the covalent attachment of SUMO to large quantities of proteins. In brief, the binding of SUMO to protein lysine is achieved by the SUMO E1, E2, and E3 enzymes: the SUMO E1 uses ATP hydrolysis to covalently connect SUMO to its active site cysteine and then transfer SUMO to the active site of the E2. The E2 further transfers SUMO onto substrates with the help of a SUMO E3^9^. SUMOylation is a highly dynamic process in which deSUMOylation is catalyzed by SUMO-specific proteases or SUMO isopeptidases^10^. Six sumo specific proteases (SENP1, 2, 3, 5, 6 and 7) are involved in the maturation and deconjugation of SUMO moieties in mammals. Biochemical assays show that SENP6 and SENP7 are most adaptive at deconjugation of SUMO2/3 chains^11^. In the present study, we selectively knocked down SENP7 in Raw 264.7 cells by lentiviral-delivered shRNA. The results showed that the SENP7 expression level in the normal group was about 3 times than that in the SENP7^shRNA^ group.

Pyroptosis is a programmed cell lytic death caused by inflammasomes. GSDMD protein is the key pyroptosis substrate of inflammatory caspases^12,13^. GSDMD is cleaved by caspases into a 31 kDa N-terminal fragment and a 22 kDa C-terminal fragment, and the N terminus is capable of triggering pyroptosis^14^. The cell membrane can be targeted and permeabilized by the N terminal fragment. Therefore, it represents a novel class of pore-forming proteins^15–17^. In the present study, LPS + ATP were applied to treat the Raw 264.7 cells to induce pyroptosis. The expressions of GSDMD, IL-1β and IL-18 were detected to evaluate pyroptosis. The immunofluorescence assay results showed that GSDMD migrated to cytomembrane accompanied by the numbers of Raw 264.7 cells decreased. The phenomenon was inhibited by SENP7 knockdown. In addition, the expression levels of GSDMD, IL-1β and IL-18 levels were all attenuated by SENP7 knockdown. These results confirmed the specific morphological features of pyroptosis and the core role of GSDMD in the pyroptosis. They also indicated that SENP7 knockdown confers a protective effect on pyroptosis.

Pyroptosis is trigged by the activation of inflammasomes via canonical and noncanonical pathways. In this study, the expression levels of NLRP3 and caspase-1 were decreased in SENP7^shRNA^ group, indicating that the activation of NLRP3 inflammasome could be inhibited by SENP7 knockdown.

There are two sequential steps (priming and activation) involved in activation of the NLRP3 inflammasome and subsequent production of bioactive IL-1β^18^. Toll-like receptors activated by recognition of danger-associated molecular patterns or pathogen-associated molecular patterns is essential to the priming course, leading to activation of NF-κB and increased synthesis of NLRP3^19^. NF-κB is the critical transcriptional factor that regulating NLRP3 synthesis^20^. The NF‐κB pathway is comprised of a dimeric transcriptional factor (NF-κB/Rel), an upstream IκB kinase (IKK) and an interacting inhibitor [inhibitor of NF-κB (IκB)]. Activated IKK phosphorylates IκB, which degenerates IκB and releases NF-κB to enter the nucleus to increase or decrease specific target gene expression^21^. In the present study, IκBα degradation and phosphorylation of NF-κB P65 subunit were inhibited by SENP7 knockdown, as well as the downstream inflammatory factors TNF-α and IL-6. This result demonstrated that the activation of NF-κB signaling pathway could be attenuated by SENP7 knockdown.

SENP7 is a SUMO-specific protease, which preferentially acts on SUMO-SUMO chains. Barry Rachael and colleagues reported that depletion of SENP7 suppresses NLRP3-dependent ASC oligomerisation, caspase-1 activation and IL-1β release, SUMOylation of NLRP3 restrains inflammasome activation^8^. The effect of SENP7 depletion on NLRP3 inflammasome was proved by our study. But in the present study, the upstream signaling pathway of NLRP3 inflammasome (NF-κB signaling pathway) was also inhibited by SENP7 knockdown. The finding indicated that NLRP3 is not the only target of SUMOylation. Over SUMOylation of other proteins might be related to the effect of SENP7 knockdown on pyroptosis, since more than 3000 sumoylated proteins have been identified^22^. However, the underlying mechanism of how SUMOylation functions need to be explored further.

In conclusion, SENP7 knockdown can inhibit the pyroptosis, the activation of NF-κB signaling pathway and NLRP3 inflammasome in Raw 264.7 cells, the mechanism may be associated with the over SUMOylation of proteins induced by SENP7 knockdown.
